# DNA and RNA base editors can correct the majority of pathogenic single nucleotide variants

**DOI:** 10.1038/s41525-024-00397-w

**Published:** 2024-02-26

**Authors:** Ariel Dadush, Rona Merdler-Rabinowicz, David Gorelik, Ariel Feiglin, Ilana Buchumenski, Lipika R. Pal, Shay Ben-Aroya, Eytan Ruppin, Erez Y. Levanon

**Affiliations:** 1 Mina and Everard Goodman Faculty of Life Sciences, Bar-Ilan University, Ramat Gan, Israel; 2 The Institute of Nanotechnology and Advanced Materials, Bar‐Ilan University, Ramat Gan, Israel; 3 Cancer Data Science Lab, Center for Cancer Research, National Cancer Institute, National Institutes of Health, Bethesda, MD USA; 4 Skip Therapeutics Ltd, 2 Ilan Ramon St, Ness Ziona, Israel

**Keywords:** Genome informatics, Data processing

## Abstract

The majority of human genetic diseases are caused by single nucleotide variants (SNVs) in the genome sequence. Excitingly, new genomic techniques known as base editing have opened efficient pathways to correct erroneous nucleotides. Due to reliance on deaminases, which have the capability to convert A to I(G) and C to U, the direct applicability of base editing might seem constrained in terms of the range of mutations that can be reverted. In this evaluation, we assess the potential of DNA and RNA base editing methods for treating human genetic diseases. Our findings indicate that 62% of pathogenic SNVs found within genes can be amended by base editing; 30% are G>A and T>C SNVs that can be corrected by DNA base editing, and most of them by RNA base editing as well, and 29% are C>T and A>G SNVs that can be corrected by DNA base editing directed to the complementary strand. For each, we also present several factors that affect applicability such as bystander and off-target occurrences. For cases where editing the mismatched nucleotide is not feasible, we introduce an approach that calculates the optimal substitution of the deleterious amino acid with a new amino acid, further expanding the scope of applicability. As personalized therapy is rapidly advancing, our demonstration that most SNVs can be treated by base editing is of high importance. The data provided will serve as a comprehensive resource for those seeking to design therapeutic base editors and study their potential in curing genetic diseases.

## Introduction

Most inherited diseases are caused by a single nucleotide variant (SNV) in the genome sequence^1^. Such a small change can corrupt the generated protein, for instance, by an incorrect amino acid (AA) translation, a misplaced termination, or a splicing error. This major group of diseases includes common medical conditions alongside a long list of low-frequency and rare diseases. The past decade has seen the rapid accumulation of knowledge regarding the genetic basis of these diseases, driven by advances in genomic sequencing technologies and Big Data analytic abilities. This has been accompanied by a budding shift in medicine from a view of genetic diseases as a permanent condition to one that envisions the era of genome editing as the harbinger of the ability to reprogram the genetic code to eliminate genetic aberration. The great advantage of base editing tools is that they can revert an SNV to the correct nucleic acid with high on-target efficiency. Indeed, this novel bundle of technologies is taking its first steps in the medical world and showing some promising results for various common genetic diseases and conditions. Base editing is also a powerful tool for realizing the era of personalized therapy, as the programable guide component of the base editor (BE) can be tailored to cater to each patient’s unique genetic features, including those with rare genetic diseases.

In general, a BE can be designed to target either the DNA sequence or the RNA sequence^2,3^. DNA editing is permanent and can potentially cure a genetic disease, but this form of editing is irreversible and, therefore, poses risks if an editing error occurs. In contrast, RNA editing is transient, as RNA molecules are constantly created and degraded in the cell. Hence, modifying the RNA sequence is easier to fine-tune and safer, as the original genomic information at the DNA level remains unchanged. Furthermore, in various genetic conditions, modifying a defined portion of the cellular mRNA to correct only some of the defective proteins is sufficient to achieve proper functionality. The drawback, however, is that it mandates continuous patient treatment.

BEs require a derivative of a deaminase, an enzyme that converts a nucleotide into another by deamination, in particular, adenosine (A) to inosine (I), with I interpreted by most cellular machineries as guanosine (G), or cytosine (C) to uridine (U), which is subsequently transformed to thymine (T). DNA BEs are composed of three fused elements: a deaminase, a Cas9 nuclease, and an associated guide RNA (gRNA) to confer target sequence specificity by Watson–Crick base pairing to the desired region^3^. Some remarkable achievements have already been reported in-vivo using this technique^4–7^. In the same manner, RNA BEs use a Cas13 nuclease and a derivative of the native adenosine deaminases acting on RNA (ADARs), an evolutionarily conserved family of editing enzymes that is responsible for the massive A-to-I endogenous editing activity in metazoan^8–10^. Intriguing works were reported using this technique^11^, as exemplified by the pioneering research in cystic fibrosis^12^. Another approach possible in RNA base editing is to design a gRNA that attracts the cell’s endogenous ADAR to target the specified nucleotide. The advantage of the latter approach is that it requires neither an external nuclease nor a programmed deaminase, thus enabling a significant reduction in the molecular size of the payload, as solely the oligonucleotide is delivered to the targeted cell; according to recent research, a guide in the length of a few dozen nucleotides is sufficient for this purpose^13^. Such a small molecule can be introduced to the tissue following chemical modifications, negating the need to insert vehicles such as viral vectors or plasmids into the body. Some successes here have been reported both in vitro^14,15^ and in vivo^16–18^.

Altogether, BEs can only edit A·T-to-G·C and C·G-to-T·A (DNA level) or A-to-I(G) and C-to-U (RNA level). Thus, directing the BE to the mutant nucleotide is possible only for G>A and T>C SNVs. However, due to the base-paired structure of the DNA molecule, directing a DNA BE to the complementary strand should likewise lead to the conversion of an erroneous nucleotide by the cellular DNA repair response^19^. Therefore, according to the Watson–Crick base-pairing rules, C>T and A>G SNVs can also be amended by this approach. This dramatically expands the number of variants that could be corrected, as C>T is the most frequent mismatch in the human genome. Despite the rapid progress in the field, the main challenge remains the BE selectivity and the concerns regarding adverse effects due to the unintentional editing of off-target sites^20,21^.

The full scope of applicability of base editing to the treatment of inherited diseases by each of these techniques is yet to be explored. In this paper, we examine the compatibility of base editing to all SNVs reported to cause human pathologic genetic conditions and present all the editing options for each relevant variant, including possible off-target sites in the genome. For variants located in coding regions that cannot be reverted, we present a novel approach that examines each deleterious AA and calculates the editing options for its replacement by a third AA that improves the translated protein, despite being different from the original reference protein. In total, we show that BEs can correct 59% of the pathogenic SNVs, and 4% can be improved, emphasizing the potential of base editing in medical genetics.

## Results

**Fig. 1:**
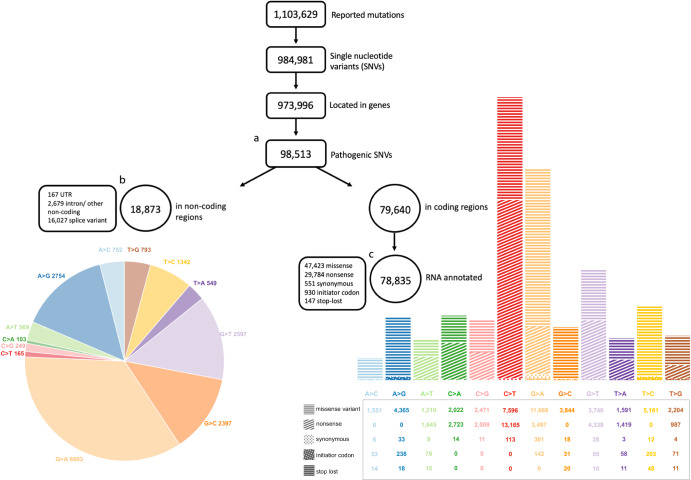
Visualization of the mutations reported in ClinVar and utilized in our analysis, displayed based on mismatch type and molecular consequences. **a** The 98,513 pathogenic SNVs located in genes. This set was utilized in our analysis of DNA base-editing. **b** The 18,873 pathogenic SNVs located in genes’ non-coding regions. This subset was utilized in our analysis of RNA base-editing in non-coding regions. **c** The 79,640 pathogenic SNVs located in genes’ coding regions, out of which 78,835 were annotated by the RNA sequence reference. This subset of 78,835 SNVs was employed in our analysis of RNA base editing within coding regions and in the assessment of amino acid improvements.

**Fig. 2:**
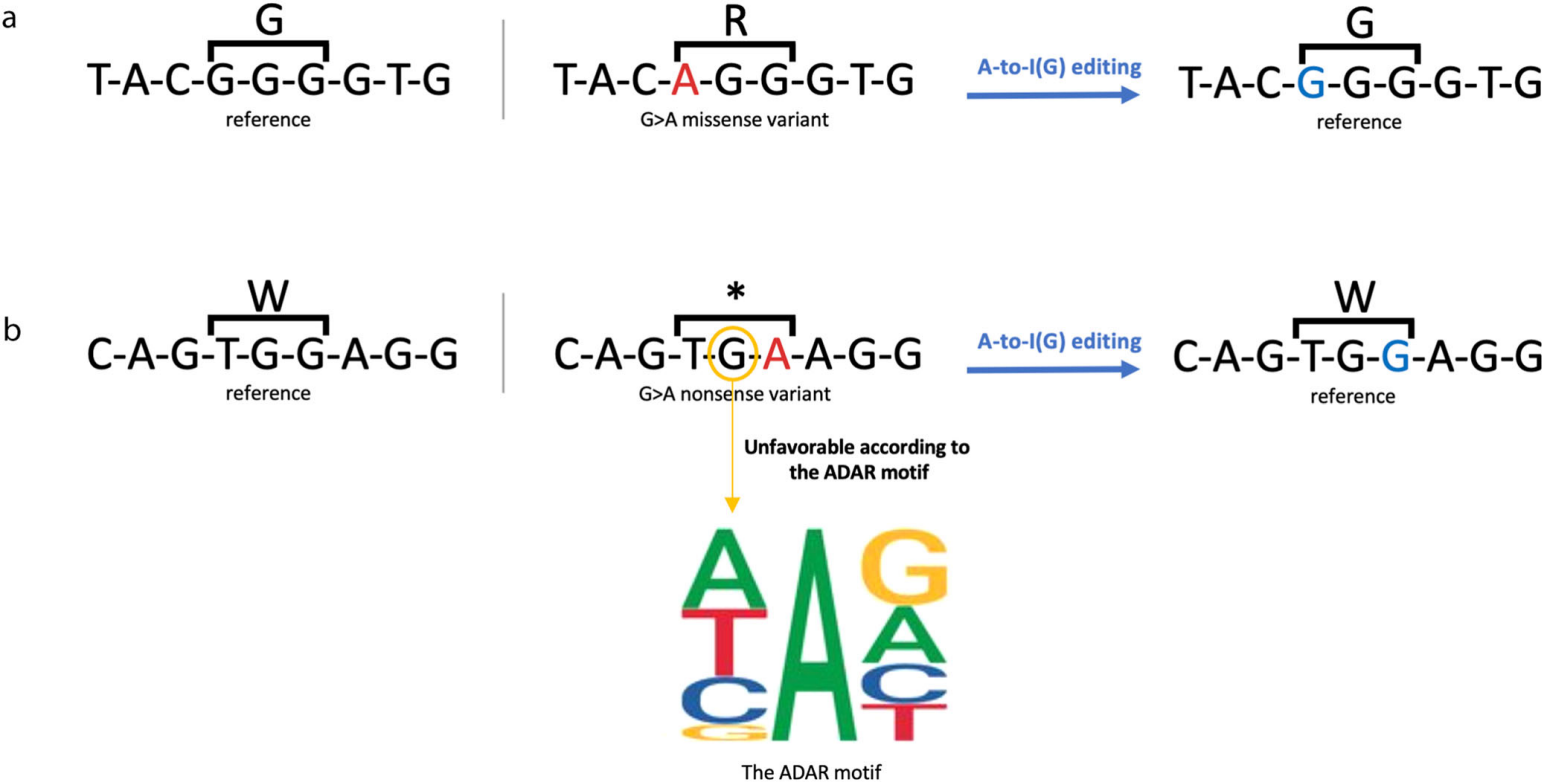
Two examples of common pathogenic SNVs that could be corrected by direct A-to-G editing. **a** The known missense variant causing achondroplasia syndrome (FGFR3):c.1138G>A (p.Gly380Arg). Direct base editing could revert the mutant A to a G. **b** The most common nonsense variant among Ashkenazy Jews causing severe cystic fibrosis (CFTR): c.3846G>A (W1282X). Direct base editing could revert the mutant A to G, thereby converting the stop codon to tryptophan (W). However, G nucleotide 5’ to the edited A may challenge this process, as can be gleaned from the ADAR motif.

We downloaded the ClinVar^22^ database, which included 1,103,629 mutations, of which 984,981 were SNVs. Of these SNVs, 973,996 were located in genes; only 98,513 were reported to be pathogenic. The distribution of the SNVs used in our analysis is shown in Fig. 1. To further illustrate the variant-correcting potential of the base editing approach, two examples of well-known pathogenic SNVs in severe genetic diseases are shown in Fig. 2. The first is achondroplasia, the most common cause for marked short stature (dwarfism). One of the most frequent missense variants is c.1137G>A on the FGFR3 gene. Whereas the reference sequence is GGG, which is translated to glycine, the mutant sequence is AGG, which is translated to arginine. Therefore, correcting the variant is possible by direct A-to-I(G) editing.

A-to-I(G) editing can also be leveraged to correct nonsense variants, such as in the second example provided, concerning cystic fibrosis (CF). This multisystemic disorder is manifested as a defect in the ion transporter encoded by the CFTR gene. Over a thousand mutations in the CFTR gene were described worldwide, and the most frequent one among Ashkenazy Jews is the stop variant W1282X. Whereas the reference sequence is TGG, which is translated to tryptophan, the mutant sequence is TGA, which results in a stop-codon. Reverting this stop codon to tryptophan is possible by direct editing. In this case, however, the editing process is more complicated if the endogenous ADAR enzyme is recruited since the ADAR motif requires the absence of a G 5’ to the edited A.

### Pathogenic SNVs that could be amended by RNA base editing

**Fig. 3:**
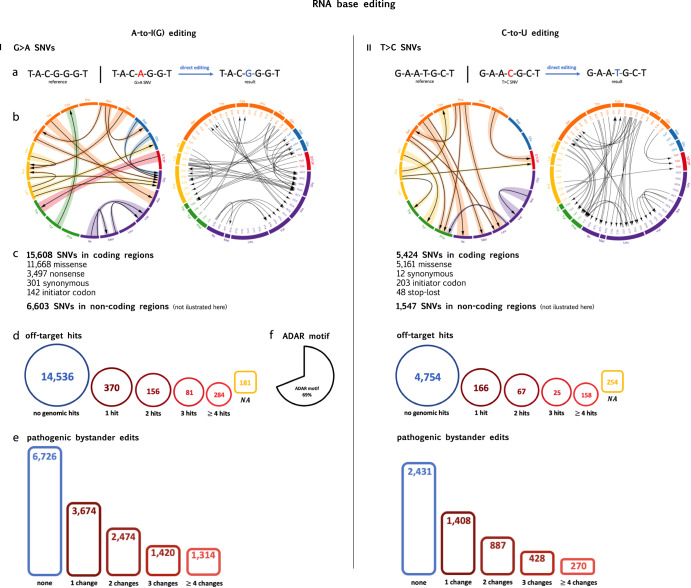
All direct base-editing possibilities at the RNA level. I: RNA A-to-I(G) base-editing, as a method to revert G>A SNVs. **a** An arbitrary example. **b** All possible amino-acid substitutions by A-to-I(G) editing. The amino acids are presented according to their chemical properties (purple = nonpolar, aliphatic R groups; green = nonpolar, aromatic R groups; yellow = positively charged R groups. orange = polar, uncharged R group, blue = negatively charged R group; red = termination). The left circos presents all the possible substitutions according to the amino acids, and the right circos presents the same data according to the codons. **c** The amount of G>A pathogenic SNVs that could be corrected by RNA A-to-I(G) base editing, presented according to molecular consequence. **d** The distribution of these SNVs based on their number of detected off-target hits at the RNA level. **e** The distribution of these SNVs based on their number of bystander changes predicted to be likely pathogenic. **f** The percentage of SNVs in which the ADAR motif is detected. II: RNA C-to-U base-editing, as a method to revert T>C SNVs.

RNA base-editing techniques are usually designed to target mRNA sequences in the cytoplasm and are suitable for targeting variants located in the coding areas of the genes. Of the 78,835 pathogenic SNVs in exons, 21,032 were suitable for direct editing: 15,608 G>A and 5424 T>C SNVs. Fig. 3 depicts all the direct editing manipulation possibilities and data regarding the variants suitable for A-to-I(G) or C-to-U editing. As is evident, 3497 nonsense variants can be reverted by A-to-I(G)editing. The findings regarding A-to-I(G) editing are pertinent to both Cas-13 and endogenous-ADAR BEs, whereas C-to-U editing can only be applied to the former.

When designing an RNA BE, an associated gRNA is programmed to confer target specificity, by base pairing to the targeted sequence. The main concern is gRNA binding to other highly identical targets, resulting in undesired off-target changes. In our search for off-target genomic regions that resemble the nucleotides surrounding the variant—representing the area of adhesion for the programmed gRNA—we constructed a sequence query encompassing the 40 bases surrounding the variant. Using the human BLAT^23^ program, we aligned the query to the RNA reference. All hits with 85% identity and 20 alignment lengths were deemed off-target sites. We found that for 91% of the G>A and C>T SNVs, zero potential off-target sites were detected, indicating that they are safe therapeutic targets regarding this manner.

Other concerning off-target changes can theoretically occur in proximity to the edited nucleotide; Once the deaminase approaches its target, it can unintentionally edit other nucleotides of the same type in a very close area. To tackle this issue, we adopted a rigorous method, concentrating on the 20 nucleotides surrounding the variant. We examined the number of potential editable nucleotides in this area and assessed the projected impact on the resulting protein following such modifications. These assessments were based on predictions provided by the AlphaMissense project^24^ for each possible nucleotide alteration. Based on their predictions, the analysis includes the number of potential bystander edits per variant that are likely to be pathogenic. For the G>A SNVs located in coding regions, an average of 4.6 surrounding A nucleotides were identified, of them 1.2 on average were anticipated to have a pathogenic impact. Notably, in 6726 variants (43%), no likely pathogenic effect was observed. In the same manner, for the T>C SNVs, 5.3 surrounding C nucleotides were found on average, and only 1.0 on average were anticipated to be pathogenic. In 2431 (45%) no likely-pathogenic effect was observed.

For 69% of the G>A SNVs, an ADAR motif, that prefers the absence of a G 5’ to the edited A was found. Therefore, these are suitable targets for BEs based on an ADAR enzyme.

RNA base editing techniques can be further expanded to target RNA sequences in the nucleus. For instance, by leveraging the endogenous ADAR p110 isoform, which is abundant in the nucleus. This expands the scope of variants that can be corrected at the RNA level since introns and other non-coding regions are transcribed at the nucleus as well. An analysis of the 18,873 pathogenic variants that are located in genes, but not in exons, identified another 7945 variants that are suitable for direct editing: 6603 G>A and 1342 T>C SNVs. In 58% of the G>A variants an ADAR motif was found (Fig. 3).

### Pathogenic SNVs that could be amended by DNA base editing

All the abovementioned G>A and T>C SNVs that are potential therapeutic targets for RNA BEs, either in the cytoplasm or in the nucleus, could also be targeted by DNA BEs. Since the BE is directed to the error nucleotide and reverts it to the reference one, we refer to this approach as *direct editing*. However, unlike RNA BEs, DNA BEs can also correct C>T and A>G SNVs by targeting the complementary strand, termed *complementary editing*. As DNA BEs act in the nucleus by definition, for this analysis, we considered all C>T and A>G SNVs located in genes, regardless of whether they are located inside or outside exons.

**Fig. 4:**
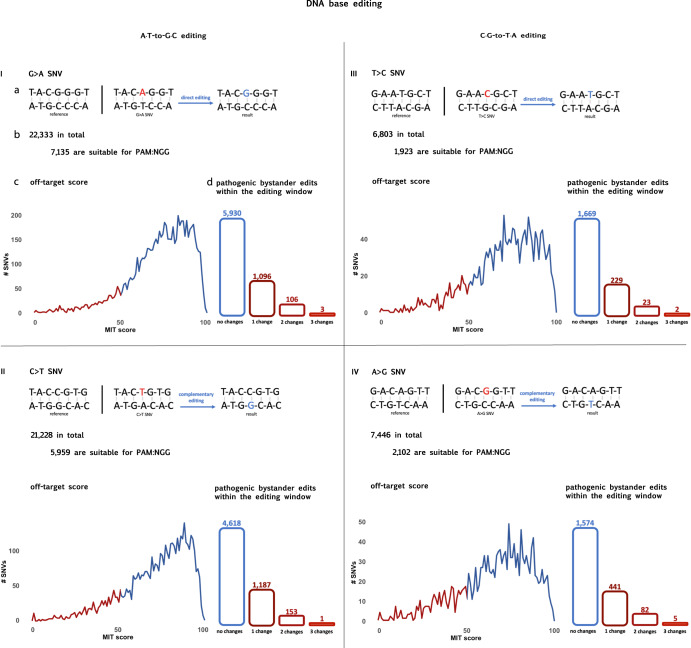
All direct and complementary base-editing possibilities at the DNA level. I: Direct A·T-to-G·C base-editing, as a method to revert G>A SNVs. **a** An arbitrary example. **b** The amount of G>A pathogenic SNVs that could be corrected by DNA A·T-to-G·C base-editing, and the subset of those possessing NGG PAM sequences. **c** The distribution of this subset of SNVs based on their MIT specificity score, which summarizes all genomic off-targets into a single numerical value. A score above 50 is indicative of a unique sequence and is considered acceptable for therapeutic purposes. **d** The distribution of this subset of SNVs based on the count of bystander changes within the NGG PAM editing window, with a focus on those predicted to be likely pathogenic. II: A·T-to-G·C base-editing directed at the complementary strand, as a method to revert C>T SNVs. III: Direct C·G-to-T·A base-editing, as a method to revert T>C SNVs. IV: C·G-to-T·A base-editing directed at the complementary strand, as a method to revert A>G SNVs.

Figure 4 depicts all the direct and complementary editing possibilities and data regarding the variants relevant to each at the DNA level. We identified 22,333 G>A and 6803 T>C SNVs suitable for direct editing and 21,228 C>T and 7446 A>G SNVs suitable for complementary editing.

When designing a DNA BE, it is essential to consider the presence of the desired protospacer/PAM sequence near the targeted nucleotide. Numerous motifs are available, based on the specific Cas9 variant in use.

In this study, we focused on the most common PAM motif generated from *Streptococcus pyogenes* (PAM: NGG), which is required to be present 12–16 bases away from the target nucleotide^25^. The motif was present in 17,119 (30%) of the editable SNVs (specifically in 32% of G>A, 28% of T>C, 28% of C>T, and 28% of A>G variants). It is important to remember that many other motifs are being developed and used, thus these numbers underestimate the actual potential of DNA BEs.

Concerning bystander edits, the calculation again depends on the chosen PAM and its associated window. We found that for the G>A SNVs in which the NGG motif is present, an average of 0.9 surrounding A nucleotides were identified in the respective 5-base editing window, of which 0.2 on average was anticipated to have a pathogenic impact. For the T>C SNVs, 1.0 surrounding C nucleotides were found on average, and 0.1 on average were anticipated to be pathogenic. In the same manner, for the C>T SNVs the numbers were 0.6/0.2 respectively, and for the A>G SNVs 1.2/0.3, respectively.

Regarding distant off-target sites, we referred to the UCSC Genome Browser CRISPR track (“CRISPR/Cas9 Sp. Pyog. target sites”)^26^, which utilizes the CRISPOR prediction tool for MIT specificity score^27,28^. This score summarizes all CRISPR/Cas9 genomic target sites into a single number ranging from 0 to 100. A guide with an MIT score above 50 is recommended for ensuring off-target safety. Out of the 17,119 variants, a score above 50 was detected in 13,183 (77%).

Table S1 in the supplementary section summarizes all ClinVar pathogenic SNVs including our added data regarding the relevant editing options, bystander and off-target hits, and ADAR and NGG motif detection.

### Base editing opportunities when reverting the pathogenic SNV is not possible

**Fig. 5:**
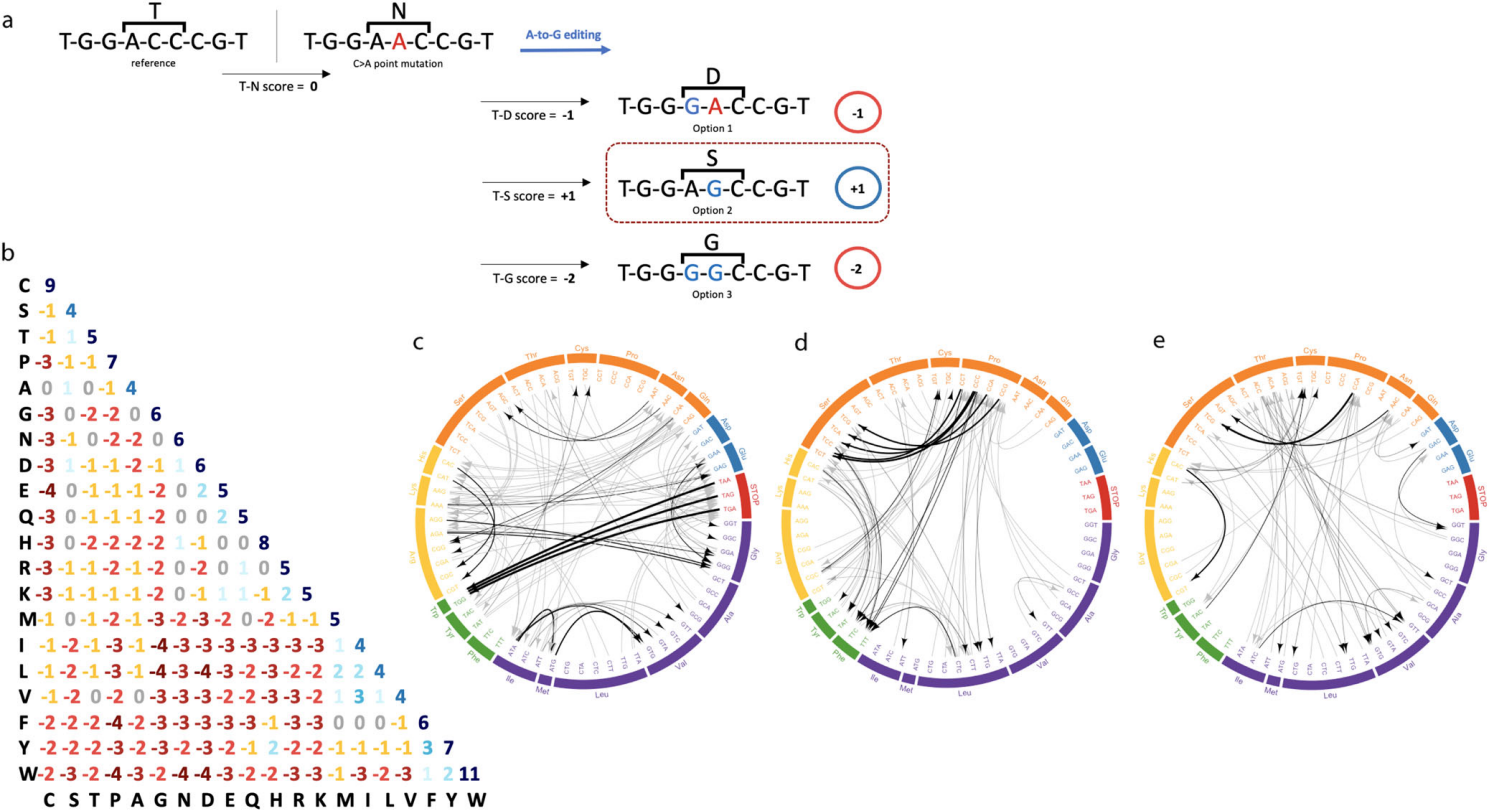
The improvement algorithm in combination with the BLOSUM62 substitution matrix. **a** An example of a variant improvement by the algorithm. Base-editing cannot revert the C>A SNV. However, by applying A-to-G editing, the mutant codon can be edited in three different ways, each of which results in a different amino acid. The algorithm chooses the option that leads to the amino acid with the highest score according to the BLOSUM62 substitution matrix. **b** The known BLOSUM62 substitution matrix. **c**–**e** All amino acid substitution possibilities according to the algorithm for the cases of A-to-G editing only (**c**), C-to-T editing only (**d**), and A-to-G and C-to-T editing in the same BE (**e**). A gray arrow represents an alteration from the reference codon to the mutant codon. A black arrow represents the best editing option from the mutant codon to the novel codon. The thickness of the black arrow correlates to the difference between the novel score and the mutant score.

**Fig. 6:**
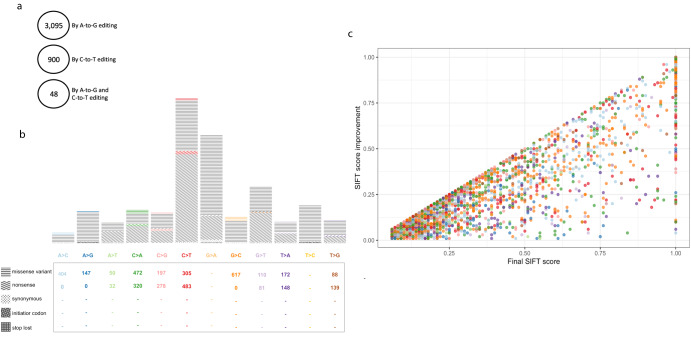
The improvement algorithm in combination with the SIFT prediction tool. The 4043 pathogenic variants that could be improved by the algorithm based on the SIFT score are presented according to the editing technique taken (**a**) and the mismatch type and molecular consequence (**b**), as is their score improvement by the post-editing score, colored according to the mismatch type (**c**).

Since 10 of the 12 mismatch types cannot be corrected by direct editing, a different approach is required. We suggest that the phenotype of a pathogenic disease could be improved by substituting the mutant (deleterious) AA with a novel AA that is more similar, though not identical, to the reference AA. This could be achieved by base editing the codon that encodes for the mutant AA. Guided on this assumption, we scanned all the possible options for editing any of the three nucleotides that encode for a given mutant AA, as well as all the options for editing more than one nucleotide of the same codon. (Of note, as the natural RNA editing mechanism tends to appear in clusters, cases of endogenous A-A or A-A-A editing are abundant, indicating that deamination of more than one Adenosine is highly feasible). For example, as shown in Fig. 5, by using A-to-G editing, a mutant codon AAC could be edited to GAC, AGC or GGC. We calculated for each deleterious AA the best option based on an AA substitution prediction tool (see the “Methods” section). See Fig. 5 for an illustrative example of applying the BLOSUM62 substitution matrix^29^ and all the possible editing options. In our AA-improvement analysis, we used the sorting intolerant from tolerant (SIFT)^30^ prediction tool to evaluate an AA’s substitution effect on a specified protein, by sequence homology and physical properties (Fig. 6). We investigated a total of 57,510 variants (these are all missense or nonsense non A>G or T>C pathogenic SNVs) and found 4043 variants that could be improved, though not corrected to the original reference amino acid. Of these, 3095 could be modified by A-to-G editing, 900 by C-to-T editing, and 48 by combining A-to-G and C-to-T editing. For example, the missense variant (PTEN):c.464A>C (p.Tyr155Ser) causes Cowden syndrome, an inherited condition characterized by multiple non-cancerous growths (i.e., hamartomas), due to a translation of TCT (serine) instead of TAT (tyrosine). By C-to-T editing, the deleterious codon could be turned into TTT (phenylalanine), resulting in a SIFT score of 1, indicating that this conversion is highly tolerated. A more complicated example is the missense variant (HNF1A):c.441C>A (p.His147Gln), which causes an inherited type of diabetes (maturity-onset diabetes of the young—MODY type 3). This variant changes the CAC (histidine) codon into the mutant CAA (glutamine) codon, resulting in a SIFT score of 0.05. By applying both A-to-G and C-to-T editing to the mutant codon (three editing actions at once), it could be modified to TGG (tryptophan), thereby increasing the SIFT score to 0.25.

On average, the improvement in the SIFT score was 0.22 per variant.

In the case of mutant stop-codons (either TAA, TAG, or TGA), these can only be converted to tryptophan (TGG) by direct-editing. We hypothesize, however, that this result is always preferable to pre-mature termination of the protein. For instance, the nonsense variant (NF1):c.4107C>G (p.Tyr1369Ter) causes Neurofibromatosis type1, one of the most common neurocutaneous syndromes, due to TAG (stop-codon) being translated rather than TAC (tyrosine). By A-to-G editing, the deleterious stop-codon could be modified to TGG (tryptophan), resulting in a SIFT score of 0.42. In total, 1195 nonsense variants that could be improved were found. A detailed list of each variant and the selected editing option is provided as part of Table S1 in the supplementary section.

### Many of the SNVs that can be base edited represent common genetic conditions

We identified, in total, 57,810 variants that could be corrected and 4043 variants that could be improved by base editing. We next sought to identify the most clinically relevant variants on this list, a complicated endeavor as it is dependent on determining the variants’ frequency in the population, which remains an unresolved challenge, for two main reasons. First, the general population is genetically heterogeneous, and current databases do not fully represent human genetic diversity. Second, most SNVs are very rare and, thus, are barely found when sequencing samplings of the population. As a result, such estimations are not accurate, especially if diverse populations are not represented.

Bearing this in mind and aiming to still give such an initial account, we used the ClinVar parameter of number-of-submitters who reported each variant. Although far from an accurate reflection of the real frequency of variants, a high number of submitters is an indirect indicator that a given variant is more common. We defined a threshold of at least three different submitters for a variant to be considered high.

In total, 19,079 (19.4%) pathogenic SNVs were reported by a high number of submitters. According to our analysis of these pathogenic SNVs, 4998 are located in exons and thus can be corrected by cytoplasmic RNA editing, 13,558 by DNA editing, and 707 can be improved.

Next, we investigated the frequencies reported in GnomAD for the pathogenic SNVs^31^. Since this database is known to include individuals with no apparent genetic disease, it is not designed to detect rare pathogenic SNVs. Yet, it is reasonable to assume that variants that do appear in GnomAD are likely to be more frequent, acknowledging the limitation that this holds true only for the population that has sequence data available. In total, 23,599 pathogenic SNVs had reported frequencies in GnomAD. According to our analysis, of these pathogenic SNVs, 5919 can be corrected by cytoplasmic RNA editing, 17,430 by DNA editing, and 866 can be improved.

Lastly, we investigated the list of 70 most common monogenic diseases in the population published by Apgar et al.^32^ and found 12,366 reported pathogenic SNVs for 41 disorders of these phenotypes. Our analysis indicates that of these SNVs, 2579 can be corrected by cytoplasmic RNA editing, 6545 by DNA editing, and 488 can be improved. Ranking these disorders by the percentage of SNVs that could be edited revealed the common diseases autosomal-dominant polycystic kidney disease (ADPKD), Beta-thalassemia and Brugada syndrome among the top five editable disorders. In the same manner, ranking the disorders by the percentage of SNVs that could be corrected by a cytoplasmic endogenous ADAR revealed Osteogenesis imperfecta and Congenital adrenal hyperplasia among the top 10. The table of the sorted disorders is available in the supplementary section (Table S4).

### Analysis of base editing’s suitability for the correction of liver and brain pathogenic SNVs

As of today, the base editing efforts are mainly focused on hepatic diseases, since delivering therapies directly to the liver is feasible by various approaches, based on intravenous injections. In the same manner, new approaches for targeting the brain tissue, based on intrathecal injections, are now emerging. Seeking to identify the pathogenic SNVs of relevance to hepatic and central nervous system diseases, we extracted from the genotype-tissue expression (GTEx) database the genes highly expressed in these tissues. This analysis revealed 581 genes that are highly expressed in the liver and 3242 in the brain (Tables S2, S3). In ClinVar, 4073 pathogenic SNVs were located in the 581 liver genes. According to our analysis, of these pathogenic SNVs, 961 can be corrected by cytoplasmic RNA editing, 2385 by DNA editing, and 194 can be improved. Of the 15,102 pathogenic SNVs located in brain genes, according to our analysis, 2950 can be corrected by cytoplasmic RNA editing, 8544 by DNA editing, and 703 can be improved. This further demonstrates the applicability of base editing to treating a variety of genetic diseases.

## Discussion

**Fig. 7:**
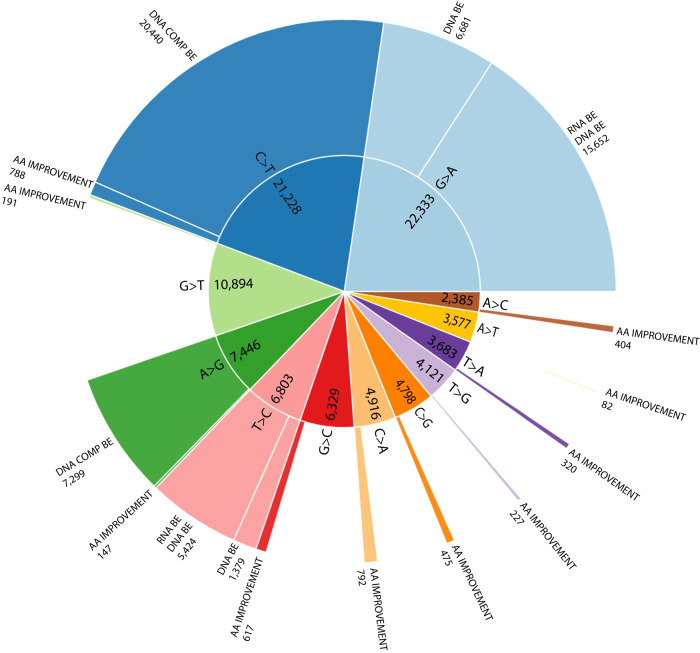
A summary of the number of variants that can be amended by base editing. The inner ring illustrates all the pathogenic SNVs located in genes, categorized by mismatch type. The outer ring represents the number and fraction of SNVs that can be amended according to the base editing technique employed. BE = base editor, COMP BE = base editor directed to the complementary strand, AA = amino acid.

This work shines a light on the copious amount of known pathogenic SNVs that can be treated by deaminases, highlighting the role programmable base editing therapies will probably assume in the near future of genetic medicine (Fig. 7). This work aligns with prior studies analyzing the ClinVar database^3,19,33^; however, here we provide a detailed exploration of the expanded potential of RNA and DNA base editing in this field.

We show that 59% of the 98,513 pathogenic SNVs located in genes can be corrected by at least one kind of BE. As a rule, DNA BEs require a programmed deaminase and act in the nucleus by definition, allowing them to correct nearly all mentioned variants, except when protospacer/PAM considerations arise. However, given the diverse motifs and adaptable proximity to the target nucleotide, the likelihood of PAM hindering BE design is low, as has been thoroughly explored in previous works within this domain^34–36^. RNA BEs that use a programmed deaminase can act either on the nuclear RNA or on the cytoplasm mRNA. With the latter option, only variants that fall in exons can be amended. Another approach is to harness the endogenous ADAR either in the nucleus (ADAR p110) or in the cytoplasm (ADAR p150), a technique that negates the need for an external deaminase and nuclease, thus reducing dramatically the molecular size of the therapy and opening up avenues for simplistic delivery approaches. The fact that an endogenous ADAR exists in the nucleus is especially interesting in the context of splicing variants since splicing regions are characterized by GT-AG sequences, indicating that A-to-I(G) corrections might be highly relevant for such variants. Indeed, numerous genetic disorders have their origin in altered splicing events, like cystic fibrosis and others^37^.

The enhanced implementation of whole-genome sequencing in the clinic and in research has resulted in the discovery of novel intergenic variants of pathogenic significance. Therefore, the numbers we report here are expected to rise further in the near future. Also, our analysis was conducted on external databases that might disproportionately represent specific populations in comparison to others. Consequently, the range of variants applicable to base editing worldwide is likely even more extensive than what our study could capture. Altogether, it is evident that base editing holds huge therapeutic potential.

The novel method we developed was able to detect an additional 4043 (4%) SNVs that could be improved by base editing directed at the mutant codon, resulting in an AA substitution. While it is reasonable to assume that the SIFT score increment reflects phenotypic improvement, one should bear in mind that, on average, the improvement in the score is 0.22, and at this point, the hypothesis is purely computational. Indeed, changing a specific AA can impair the protein’s function. For instance, A-to-G editing can convert serine, threonine, or tyrosine to glycine, alanine, and cysteine respectively, disrupting phosphorylation sites. It can also convert lysine to arginine or glycine, suppressing lysine methylation or acetylation (Fig. 3-Ib). Hence, utilizing this method warrants further biological investigation for each case individually.

Interestingly, our investigation into off-targets in the context of ADAR-based techniques revealed that, despite the substantial number of observed variants, no hits were detected for the vast majority, classifying them as safe therapeutic targets. The off-target analysis relies on a fixed number of nucleotides (40) that resemble the gRNA component, aligning with recent reports regarding the ideal guide length for such techniques. Also, we opted for a fixed identity parameter of 85%, considered conservative, as altering the range from 75% to 95% had practically no effect on the results. In theory, lowering the identity with almost no off-target “cost” enables the insertion of additional mismatches to the programmed guide, making it more selective to one allele over another in cases of mutation heterozygosity.

Of note, regarding RNA base editing, our off-target results are even stricter than in vivo, since the detected hits are not necessarily expressed in the target tissue, and even if they are, RNA editing modifies only a defined portion of the RNA molecules in a given cell. Also, a noteworthy point is that direct A-to-I(G) editing cannot result in a stop-codon (TAA, TAG, or TGA) unintentionally.

We also examined the possible bystander changes that may occur if the deaminase edits proximal nucleotides to the target and evaluated whether these changes are predicted to be deleterious. The results for DNA BEs are subject to greater dynamism depending on the specific Cas used and its associated editing window. In contrast, for RNA BEs, extensive base pair complementation is required, determining the adjacent bases that are at risk. Interestingly, a recent RNA base editing study demonstrated that the inclusion of an extra chemical compound alongside the gRNA significantly diminishes the occurrence of bystander edits^38^.

We showed that in most of the SNVs prone to A-to-I(G) editing, the 5’ nearest neighbor is not guanosine, classifying these variants as suitable targets for base editing based on the ADAR motif. Nevertheless, as for the rest of the variants, a recent paper suggests an alternative method to overcome this limitation^39^.

In this work, we excluded all mitochondrial DNA mutations. However, it is likely that in the future, as technologies evolve, such mutations will become candidates for base editing as well^40^.

Finally, it is important to acknowledge that as of today, there is a limited number of tissues to which base editing can be delivered. One of them is the liver for which several options for delivery are available. The second is the brain, for which direct injection of oligos to the cerebral spinal fluid is feasible. For both, we calculated the clinically relevant SNVs that could be targeted in highly expressed genes in these tissues, though we recognize that gene expression serves as a suggestive rather than definitive indicator for determining the clinical significance of a specific gene in a particular tissue. Nevertheless, It is important to perform forward-looking studies that aim to systematically evaluate the potential of what can be done if and once new technologies for delivering BEs in a tissue-specific manner are developed.

Identifying the particular diseases suitable for base editing presents a challenge that necessitates collaboration between scientists and clinicians. For instance, a crucial clinical factor to consider is the age at which the genetic condition manifests. On one hand, diseases that are expected to progress later in life require extended treatment durations. On the other hand, treating an early onset disease may not be beneficial if the phenotype is already evident during a very young age and is irreversible, as seen, for example, in genetic disorders impacting neurological development.

In conclusion, we show that 59% of human pathogenic SNVs can be potentially corrected by DNA base editing techniques, and 29% can be corrected by RNA base editing techniques, which may have their own translational advantages. We additionally evaluated and ranked the top genetic disorders that could potentially be treated by BEs. As genome-editing approaches are rapidly progressing, it stands to reason that a revolution in the field of genetic diseases is just around the corner. Hopefully, this work will help scientists and clinicians design successful targets for therapies, advancing the potential use of base editing for curing genetic diseases.

## Methods

### Human SNVs data

We downloaded the ClinVar database^22^ from the UCSC table browser on 08-Nov-2021. On this date, the database included 1,103,629 mutations, of which 984,981 were reported as SNVs. We excluded 147 genetic downstream and 1,067 genetic upstream transcript variants, 237 variants with no sequence alteration, 7091 that had no molecular consequence, 391 mutations that were mistakenly classified as SNVs, and 2052 mitochondrial mutations—remaining with 973,996 SNVs located in autosomal genes.

Next, we filtered the 98,513 SNVs reported to be clinically pathogenic by including any phrase that contained derivatives of the word “pathogenic” (e.g., “pathogenic”, “likely pathogenic”). In cases reported as “conflicting interpretations of pathogenicity”, the variant was included if at least one submitter reported this variant as pathogenic according to the ClinVar VCF file.

For the DNA base-editing analysis, we extracted the DNA sequence of each variant using Bedtool getfasta (hg38 reference genome). All 98,513 SNVs were included. For the RNA base-editing analysis, we used the isoform “MANE SELECT” as presented in ClinVar and the reported coordinates (column OrigName) of each variant, to extract the RNA sequence from the CDS FASTA file that was downloaded from the UCSC genome browser (http://genome.ucsc.edu)^26^ on 27-Mar-2022, as well as the reading frame of each variant (i.e., the 3-nucleotide codon sequence). We included only the 78,923 SNVs in which the data matched. For the first part of the RNA base-editing analysis, we further filtered the 78,835 SNVs located in coding regions of the genes: 47,423 missense, 29,784 nonsense, 551 synonymous, 930 initiator-codon variants, and 147 stop-lost variants (Fig. 1).

### Amino-acid improvement

We created an algorithm for all the SNVs that cannot be reverted by direct A-to-G or T-to-C editing. The algorithm calculates the best option for substituting a mutant (deleterious) codon with a novel codon (not the reference one), by leveraging A-to-G and/or T-to-C editing. Each possible new AA is scored using a validated substitution prediction tool and compared to the deleterious AA score. In case of improvement, this editing option is considered. The algorithm prefers the best score with minimum exchanges when multiple editing improvement options are possible.

For this analysis, we excluded all the irrelevant variants (i.e., synonymous, initiator-codon variants, stop-lost, and G>A and T>C SNVs).

For a descriptive purpose, we first applied the BLOSUM62 substitution matrix^29^, which scores all AA substitution-possibilities on a scale of −4 to 11. We added to the results all the possible options to substitute a stop-codon with a novel AA, as stop-codons are not included in BLOSUM62. Then, for a more clinically relevant purpose, we applied the Sorting Intolerant from Tolerant (SIFT)^30^ prediction tool, known to evaluate the substitution effect on each protein by sequence homology and physical properties. The SIFT score ranges from 0.0 (deleterious) to 1.0 (tolerated), with a score between 0.0 and 0.05 considered very deleterious. Thus, for our clinical analysis, only a SIFT score above 0.05 was considered. In the same manner, as in BLOSUM62, we included by default any stop-codon substitution with a novel AA.

### Gene expression by GTEx

The Genotype-Tissue Expression (GTEx) project was supported by the Common Fund of the Office of the Director of the National Institutes of Health, and by NCI, NHGRI, NHLBI, NIDA, NIMH, and NINDS. The data used for the analyses described in this manuscript were obtained from the GTEx Portal on 31-Jan-2023. We used this database to extract all the genes that are highly expressed (above 10 transcripts per million) in the liver but lowly expressed in all other tissues (average expression below 10 transcripts per million). The same was done for the brain. The list of genes and expression levels is available in the supplementary section.

### Reporting summary

Further information on research design is available in the Nature Research Reporting Summary linked to this article.

### Supplementary information

Supplementary Informative File

REPORTING SUMMARY

## Data Availability

All data generated or analyzed during this study are included in this published article and its supplementary information files. The ClinVar dataset, a publicly accessible repository of clinically relevant genomic variations managed by the National Center for Biotechnology Information (NCBI), was retrieved from UCSC, version November 2021, and can be accessed at https://hgdownload.soe.ucsc.edu/goldenpath/archive/hg38/clinvar/2021-11/. Proteome-wide missense variant effect predictions from the alphaMissense project were obtained from their community resource repository, which can be accessed at https://console.cloud.google.com/storage/browser/dm_alphamissense. Data from GTEx, a comprehensive resource providing insights into the relationship between genetic variation and gene expression across various human tissues, were sourced from the GTEx Portal at https://gtexportal.org/home/downloads/adult-gtex.

## Code availability

The source code used to produce the results and analyses presented in this manuscript are available on a GitHub repository at: https://github.com/arieldadush/BE-on-genetic-point-mutations/tree/main.
