# Supplementary material for: DNA and RNA base editors can correct the majority of pathogenic single nucleotide variants
Source: NPJ Genom Med. 2024 Feb 26;9:16. doi: 10.1038/s41525-024-00397-w (PMC10897195; doi:10.1038/s41525-024-00397-w)
Supplement: Supplementary file 1 — Supplementary Informative File [file 41525_2024_397_MOESM1_ESM.docx]

**Supplementary Informative File**

All supplementary tables can be downloaded from this GitHub link:

<https://github.com/arieldadush/BE-on-genetic-point-mutations/tree/main/tables%20for%20supplementary>

Table S1 : A detailed table presenting all the pathogenic SNVs reported in ClinVar, with added data for each regarding the relevant editing options, bystander and off-target hits and ADAR motif.

The table includes the columns:

orig_name – ClinVar variant full name

location – genomic position

gene – gene

strand – DNA strand [ +/- ]

mutation – SNV mismatch type

mol_conseq – molecular consequence

clin_significance – clinical significance, according to ClinVar

phenotype_list – phenotypes associated with this variant, according to ClinVar

num_submit – numbers of submitters

last_date_eval – last evaluation date by ClinVar

gnomAD_freq – Variant frequency according to gnomAD

editing_option – possible base editors for this variant [ none/ DNA direct BE/ DNA complementary BE/ RNA BE/AA improvement ]

RNA_41n_seq_for_guide – a sequence of 41 bases surrounding the variant (20 from each side) at the RNA level

RNA_off_target_hits_85_min_identity – number of off-target hits with at least 85% identity, as detected at the RNA level

count_RNA_bystander_edits – number of bases of the same type in the 21 bases surrounding the variant (10 from each side).

count_RNA_pathogenic_bystander_edits - number of bases of the same type in the 21 bases surrounding the variant (10 from each side), that are predicted to be pathogenic upon editing, based on the alphaMissense prediction tool.

ADAR_motif – existence of ADAR motif [ yes/no/not relevant ]

NGG_editing_window – the genomic location of the NGG PAM editing window. Presented on the positive strand.

MIT - the MIT specificity score for NGG PAM summarizes all genomic off-targets into a single numerical value, with a higher score indicating fewer hits [0-100]

count_DNA_bystander_edits – number of bases in the NGG PAM editing window of the same type as the variant

count_DNA_pathogenic_bystander_edits- number of bases in the NGG PAM editing window of the same type as the variant that are predicted to be pathogenic upon editing, based on the alphaMissense prediction tool

improv_new_codon – the optimal option for amino-acid improvement based on to the highest achievable SIFT score

improv_editing_tech – the editing option that should be used to achieve the improved codon, and the number of editing required (in brackets)

SIFT_score - the mutant SIFT score > the improved SIFT score

liver_expression - is this gene highly expressed in the human liver, based on GTEx [yes/no]

brain_expression – Is this gene highly expressed in the human brain, based on GTEx [yes/no]

Table S2 : Genes exhibiting significant expression levels in the liver.

Each row presents one gene and its average transcripts per million (TPM) value in the liver tissue compared to the value in all other tissues combined.

Table S3 : Genes exhibiting significant expression levels in the brain.

Each row presents one gene and its average TPM value in the brain compared to the value in all other tissues combined.

Table S4 : Monogenic diseases ranked according to the percentage of editability.

#Orphanet – the disease’s reference number according to Orphanet

Disease – the common name of the disease

Synonyms – additional names of the disease

%Editable SNVs – the percentage of reported SNVs for this disease that are suitable for base editing
